# Pathophysiologic Contributions of Visceral Adiposity to Left Ventricular Diastolic Dysfunction

**DOI:** 10.3390/jcdd10060247

**Published:** 2023-06-05

**Authors:** Reika Nagata, Masaru Obokata, Miki Matsui, Hiroki Matsui, Yuko Seki, Takamichi Igarashi, Hiroaki Sunaga, Ryo Kawakami, Tomonari Harada, Kazuki Kagami, Hiroshi Saeki, Ken Shirabe, Tatsuya Iso, Hideki Ishii

**Affiliations:** 1 Department of Cardiovascular Medicine, Gunma University Graduate School of Medicine, 3-39-22 Showa-machi, Maebashi 371-8511, Gunma, Japan; h211b013@gunma-u.ac.jp (R.N.); y_seki@gunma-u.ac.jp (Y.S.); sunaga.hiroaki@g.ashikaga.ac.jp (H.S.); r.kawakami.1526@gmail.com (R.K.); tharada@gunma-u.ac.jp (T.H.); mirror.1028k@gmail.com (K.K.); isot@gunma-u.ac.jp (T.I.); hkishii@med.nagoya-u.ac.jp (H.I.); 2 Department of Laboratory Sciences, Gunma University Graduate School of Health Science, 3-39-22 Showa-machi, Maebashi 371-8511, Gunma, Japan; miki-matsui@gunma-u.ac.jp (M.M.); hmatsui@gunma-u.ac.jp (H.M.); 3 Department of Radiology, Gunma University Hospital, 3-39-15 Showa-machi, Maebashi 371-8511, Gunma, Japan; 4 Department of General Surgery, Gunma University Graduate School of Medical Sciences, 3-39-22 Showa-machi, Maebashi 371-8511, Gunma, Japan; takamichi.iga@gmail.com (T.I.); h-saeki@gunma-u.ac.jp (H.S.); kshirabe@gunma-u.ac.jp (K.S.); 5 Division of Cardiovascular Medicine, National Defense Medical College, 3-2 Namiki, Tokorozawa 359-8513, Saitama, Japan

**Keywords:** adipokines, cytokines, heart failure with preserved ejection fraction, visceral adiposity

## Abstract

Background: Visceral fat produces inflammatory cytokines and may play a major role in heart failure with preserved ejection fraction (HFpEF). However, little data exist regarding how qualitative and quantitative abnormalities of visceral fat would contribute to left ventricular diastolic dysfunction (LVDD). Methods: We studied 77 participants who underwent open abdominal surgery for intra-abdominal tumors (LVDD, n = 44; controls without LVDD, n = 33). Visceral fat samples were obtained during the surgery, and mRNA levels of inflammatory cytokines were measured. Visceral and subcutaneous fat areas were measured using abdominal computed tomography. Results: Patients with significant LVDD had greater LV remodeling and worse LVDD than controls. While body weight, body mass index, and subcutaneous fat area were similar in patients with LVDD and controls, the visceral fat area was larger in patients with LVDD than in controls. The visceral fat area was correlated with BNP levels, LV mass index, mitral e′ velocity, and E/e′ ratio. There were no significant differences in the mRNA expressions of visceral adipose tissue cytokines (IL-2, -6, -8, and -1β, TNFα, CRP, TGFβ, IFNγ, leptin, and adiponectin) between the groups. Conclusions: Our data may suggest the pathophysiological contribution of visceral adiposity to LVDD.

## 1. Introduction

More than half of patients with heart failure (HF) have a left ventricular (LV) preserved ejection fraction (HFpEF) [1,2]. HFpEF is a substantial public health problem owing to its increasing prevalence, high morbidity and mortality, and limited treatment options [1,2]. Growing evidence suggests that systemic inflammation secondary to metabolic comorbidities plays an important role in the pathophysiology of HFpEF, leading to myocardial inflammation and fibrosis and alterations in cardiomyocyte nitric oxide–cyclic guanosine monophosphate (NO–cyclic GMP) signaling [3,4]. These conditions can promote cardiomyocyte hypertrophy, fibrosis, myocardial fibrosis, and LV diastolic dysfunction (LVDD) [3,4].

Obesity, especially when associated with increased visceral adiposity, may be a primary pathophysiologic driver in HFpEF syndrome through the production of inflammatory cytokines [4,5,6]. Indeed, visceral fat is greater in patients with HFpEF than that in non-HF controls, and HFpEF is related to activation in inflammatory markers such as tumor necrosis factor (TNF)-α, interleukin-6 (IL-6), and C-reactive protein (CRP) [6,7,8,9,10,11,12]. However, little data exist regarding how qualitative and quantitative abnormalities of visceral adiposity would contribute to HFpEF pathophysiology, particularly LVDD. We hypothesized that inflammatory cytokines and adipokines would be elevated in the visceral fat of patients with LVDD, and that the severity would be related to LVDD.

To test this hypothesis, we collected visceral adipose tissue from patients with significant LVDD or established HFpEF who underwent open abdominal surgery and examined the mRNA expressions of inflammatory markers as compared to non-LVDD controls. We also performed abdominal computed tomography (CT) to evaluate the quantitative abnormality of visceral fat and its effects on cardiac dysfunction in HFpEF.

## 2. Materials and Methods

### 2.1. Study Population

This was a prospective observational study that evaluated the qualitative and quantitative abnormalities of visceral fat in patients with LVDD or established HFpEF. We screened patients who were scheduled to undergo open abdominal surgery for an intra-abdominal tumor (regardless of malignant or benign) in our hospital between October 2019 and July 2021. From this cohort, patients with a comprehensive echocardiographic evaluation in a stable state within the previous 3 months were identified. The study was approved by our institutional review board (HS2019-152, Gunma University Hospital, Clinical Research Review Board) and was performed in accordance with the declaration of Helsinki. Written informed consent was obtained from all participants before index surgery. The de-identified participant data will not be shared. All authors have read and agreed to the manuscript as written.

Clinical demographics, past medical history, current medications, laboratory results, and echocardiographic data were collected from a detailed chart review. Significant LVDD was defined as the presence of at least one of the following: (i) LV hypertrophy (defined as LV mass index [LVMI] > 115 g/m^2^ in men and >95 g/m^2^ in women); (ii) left atrial enlargement (defined as left atrial [LA] volume index ≥ 34 mL/m^2^); (iii) abnormal E/e′ ratio (defined as the average ratio of mitral inflow peak early diastolic velocity [E] to mitral annular early diastolic tissue velocity [e′] ≥ 13); and (iv) impaired LV global longitudinal strain (GLS < 18% [absolute value]) as previously described [13]. The diagnosis of HFpEF was then defined as the presence of typical clinical symptoms of HF (exertional dyspnea, fatigue, or peripheral edema), an EF ≥ of 50%, and objective evidence of LVDD [14]. In this study, patients with LVDD and those with HFpEF were analyzed together (referred to as LVDD). Patients with EF < 50%, non-group II pulmonary artery hypertension, significant left-sided valvular heart disease (mild or greater stenosis or moderate or greater regurgitation), acute coronary syndrome, congenital heart disease, or cardiomyopathies were excluded. Ideal body weight (in kilograms) was calculated from height (in centimeters): (height − 100) − ([height − 150]/a), where a = 4 for men and 2.5 for women. Nutritional status was assessed using the Geriatric Nutritional Risk Index (GNRI) [15].

### 2.2. Visceral Fat Sampling and mRNA Measurements

**Figure 1:**
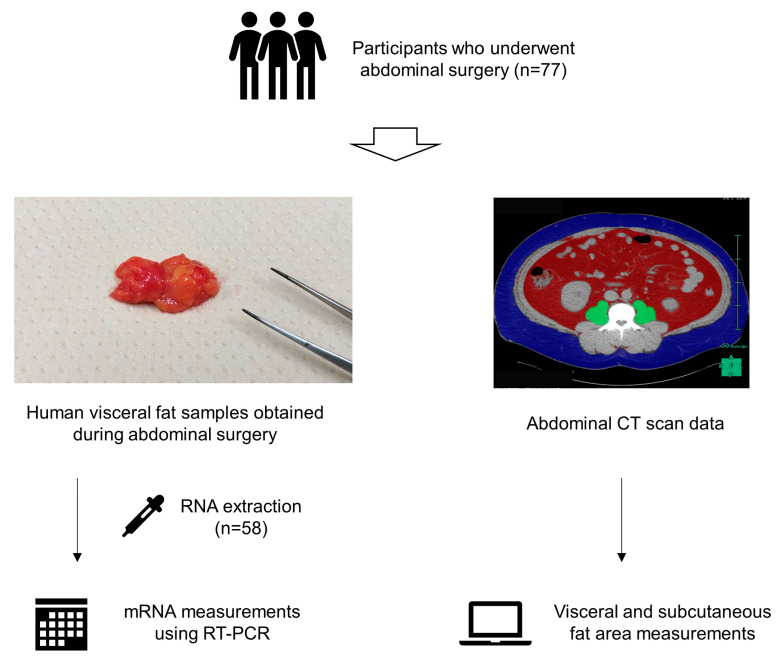
Study flow. Patients who underwent abdominal surgery for an intra-abdominal tumor (n = 77) were enrolled. Visceral fat samples were obtained during indexed surgery, and inflammatory cytokine and adipokine mRNA expression levels in the visceral fat samples were measured via real-time quantitative PCR. Visceral and subcutaneous fat areas were assessed on abdominal computed tomography (CT) data.

Visceral fat samples were obtained from a resected specimen during the operation and were stored at −80 °C until analysis (Figure 1). Among 77 participants, sufficient fat samples were not obtainable in 7 patients. Inflammatory cytokine and adipokine mRNA expression levels in visceral fat samples were measured via real-time quantitative PCR in 58 randomly selected patients due to the limited number of wells in the PCR plate (controls, n = 26 and HFpEF, n = 32). These markers included TNF-α, IL-6, IL-1β, IL-2, IL-8, CRP, transforming growth factor (TGF)-β, interferon (IFN)-γ, adiponectin, and leptin (Table S1).

Total RNA was extracted from the visceral fat samples using TRIzol regent (Thermo Fisher Scientific, Waltham, MA, USA) according to the manufacturer’s protocol. One microgram of RNA was used for reverse transcription with the ReverTra Ace qPCR RT Kit (TOYOBO, Osaka, Japan), and qRT-PCR analysis was performed using the THUNDERBIRD SYBR qPCR Mix (TOYOBO) according to the manufacturers’ protocols. qRT-PCR was carried out using a StepOnePlus real time PCR system (Applied Biosystems, Waltham, MA, USA). The SYBR Green method was used to quantify target mRNA expression. Three samples of each were measured using βactin as an internal control. To prove that the amplified PCR product was specific for the mRNA of interest, the melting curve was confirmed to show a unimodal waveform.

### 2.3. Quantitative Assessments of Visceral and Subcutaneous Fat

The visceral and subcutaneous fat areas were assessed using commercially available semi-automated software (SYNAPSE VINCENT, Fujifilm Inc., Tokyo, Japan) for all participants [16]. Unenhanced abdominal CT data were imported into the software in the Digital Imaging and Communication in Medicine format. The software automatically identified and calculated visceral and subcutaneous fat areas using a single cross-sectional image at the level of the 3rd lumbar vertebra; they were defined as the areas containing pixels with an attenuation value of −190 to −30 HU [7]. All CT measurements were performed in a blinded manner (Y.S.).

### 2.4. Cardiac Structure and Function Assessment

Comprehensive echocardiography was performed according to the contemporary guidelines using commercially available ultrasound systems (Vivid E95, GE Healthcare, Horten, Norway) [17]. LV volumes (end-diastolic volume [EDV] and end-systolic volume [ESV]), LA volume, and EF were measured using the biplane method of disks. LV diastolic function was assessed using mitral inflow velocities, mitral annular tissue velocities, and average E/e′ ratio. Right ventricular (RV) systolic pressure was calculated using the peak tricuspid regurgitation (TR) velocity and estimated right atrial pressure [17]. RV systolic function was assessed via tricuspid annular plane systolic excursion (TAPSE). Myocardial deformation analyses were performed to assess GLS and the LA reservoir and booster pump strain using commercially available software (EchoPAC PC version 204, GE, Milwaukee, WI, USA) [18]. The LV and LA strain were expressed as absolute values.

### 2.5. Statistical Analysis

Data are reported as mean (SD), median (IQR), or number (%) unless otherwise specified. Between-group differences were compared using chi-square test, unpaired t-test, or Mann–Whitney U test. Pearson’s correlation coefficient was used to assess relationships between two variables of interest, where non-normally distributed variables were first log-transformed. All tests were two-sided, with a *p*-value of <0.05 considered significant. All analyses were performed with JMP 14.0.0 (SAS Institute, Cary, NC, USA).

## 3. Results

### 3.1. Baseline Characteristics

The current study included 44 patients with LVDD (LVDD, n = 42; established HFpEF, n = 2) and 33 controls free of LVDD. Patients with LVDD were older and had a higher prevalence of coronary artery disease, atrial fibrillation, and β-blocker use (Table 1).

Most patients were found to have a malignant abdominal tumor, but its prevalence was similar in patients with LVDD and controls (77% vs. 85%, *p* = 0.41). Sex, body weight, body mass index (BMI), and the prevalence of other comorbidities were similar between the groups. Serum levels of B-type natriuretic peptide (BNP) and creatinine were higher, and albumin levels were lower in patients with LVDD compared to those in controls. The GNRI did not differ between groups.

Compared to controls, patients with LVDD displayed a larger LV diastolic dimension, greater LVMI and relative wall thickness, and worse diastolic function, with a lower mitral e′ velocity, higher average E/e′ ratio, larger LAVI, and lower LA reservoir strain (Table 2). The LV systolic function was more impaired in patients with LVDD than in controls, as evidenced by lower mitral s′ velocity and GLS. There were no differences in right heart function and pressures between the groups.

### 3.2. Quantitative and Qualitative Assessments of Visceral Adiposity

**Figure 2:**
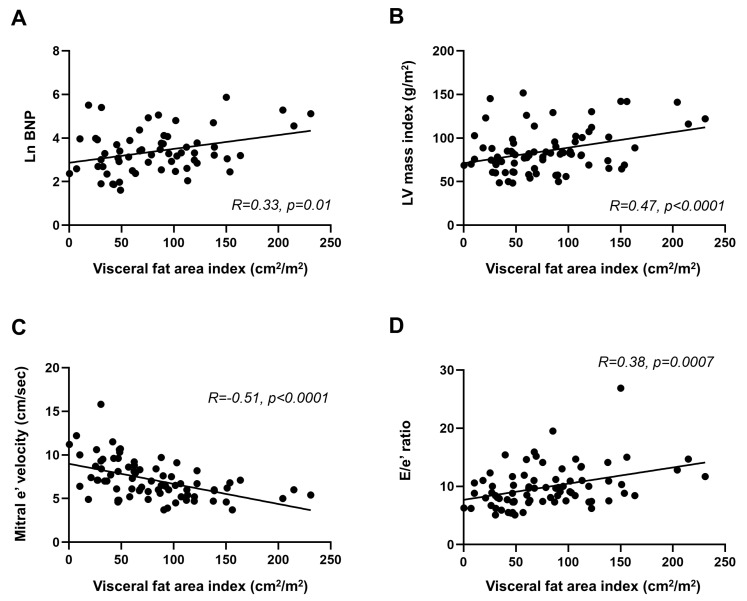
Correlations of visceral adipose tissue area with LV diastolic dysfunction. Visceral fat area index was correlated with (**A**) B−type natriuretic peptide (BNP) levels, (**B**) left ventricular (LV) mass index, (**C**) early diastolic mitral annular velocity (e′), and (**D**) the ratio of early diastolic mitral inflow velocity/e′.

Despite the similar body weight and BMI as well as CT-derived subcutaneous fat area between the groups, patients with LVDD demonstrated a larger visceral fat area on the abdominal CT scan than the controls, and this difference remained significant even after indexing for body surface area (Table 3). Among all participants, the visceral fat area index was related to worse LV diastolic function, with higher BNP levels, larger LVMI, lower mitral e′ velocity, and higher E/e′ ratio (Figure 2).

**Figure 3:**
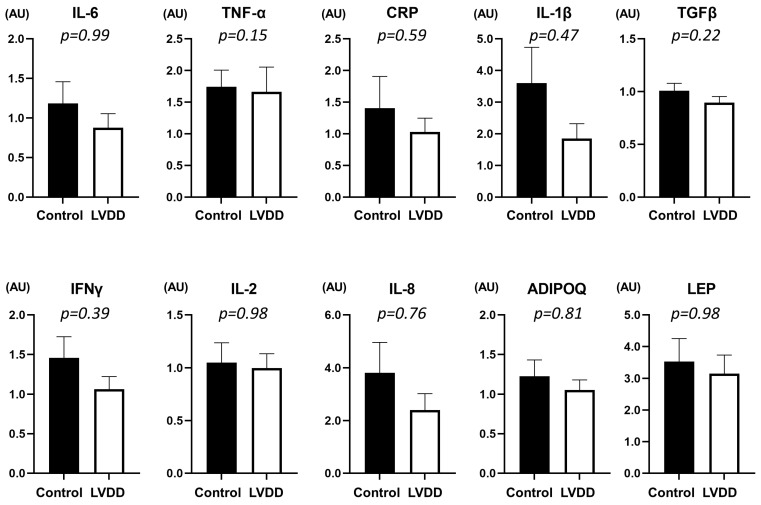
Comparisons of relative mRNA expressions of inflammatory cytokines between LVDD and controls. Values represent mean ± SE. The inflammatory cytokine and adipokine mRNA expressions did not differ between patients with LVDD and controls (all *p* > 0.1). ADIPOQ, adiponectin; CRP, C-reactive protein; HFpEF, heart failure with preserved ejection fraction; IFN, interferon; IL, interleukin; LEP, leptin; TGF, transforming growth factor; and TNF, tumor necrosis factor.

Figure 3 shows mRNA expressions of visceral adipose tissue inflammatory genes in patients with LVDD and controls. There were no significant differences in inflammatory cytokine and adipokine mRNA expressions between the groups, while levels of TNF-α and IFN-γ were modestly correlated with lower mitral e′ velocity (r = −0.31, *p* = 0.02 and r = −0.30, *p* = 0.03). The inflammatory cytokine and adipokine mRNA expressions did not differ between participants with and without a malignant tumor (all *p* > 0.1).

## 4. Discussion

To the best of our knowledge, we for the first time collected visceral adipose tissue from patients with LVDD or HFpEF to examine mRNA expressions of inflammatory cytokines and adipokines in LVDD/HFpEF as compared to controls free of LVDD. We also performed abdominal CT to quantify the visceral fat area and its association with cardiac structure and function in LVDD. Despite the similar subcutaneous fat area and BMI, patients with LVDD demonstrated a significantly larger visceral fat area than the controls, and the visceral fat area index was related to worse LVDD. We found no differences in inflammatory cytokine and adipokine mRNA expressions in patients with LVDD and the controls. The current preliminary data may provide insights into the pathophysiological roles of quantitative abnormalities of visceral adiposity in HFpEF.

### 4.1. Visceral Obesity in the HFpEF Inflammatory Paradigm

The current paradigm suggests that systemic inflammation caused by cardiac and metabolic comorbidities is responsible for LV hypertrophy, stiffening, and LVDD, contributing to the underlying pathophysiology of HFpEF [3]. Obesity is considered to be the key comorbidity generating systemic inflammation in HFpEF [12,19]. Growing evidence has demonstrated the pathologic importance of regional adiposity, especially visceral adipose tissue in patients with HFpEF [7,20,21]. Visceral adiposity is associated with chronic low-grade systemic inflammation through upregulation of proinflammatory adipokines (e.g., leptin, TNF-α, IL-6, and resistin) and downregulation of anti-inflammatory adipokines (e.g., adiponectin and omentin-1) [22]. Although it has been reported that visceral fat is greater in patients with HFpEF than that in non-HF controls [7], no study has investigated qualitative abnormalities of visceral fat in patients with HFpEF.

In the current study, we found no differences in the mRNA expressions of inflammatory cytokines (TNF-α, IL-6, IL-1β, IL-2, IL-8, CRP, TGF-β, IFN-γ) and adipokines (adiponectin and leptin) between patients with LVDD/HFpEF and controls free of LVDD. Several factors might contribute to this result. In accordance with previous studies examining Japanese patients with HFpEF [23,24,25,26], prevalence of obesity (BMI ≥ 25 kg/m^2^) was low in this study (29%). The lower rate of obesity might have affected the lower mRNA expressions of inflammatory cytokines and adipokines. The current study included patients who underwent abdominal surgery for intra-abdominal tumors. Although the prevalence of malignancy did not differ between the groups, the presence of a malignant tumor might influence the mRNA expressions of visceral adipose tissue inflammatory genes. Most of the patients with LVDD in this study were asymptomatic, and this also might influence the overall results. Because of these limitations, we cannot exclude the possibility that visceral adiposity contributes to inflammatory states in HFpEF. [3,19] Further study is required to determine the pathophysiologic role of qualitative abnormalities of visceral adiposity in patients with HFpEF, especially those with obesity.

### 4.2. Contribution of Quantitative Abnormalities of Visceral Fat to LVDD

Recent studies have demonstrated the pathophysiologic significance of the accumulation of visceral adipose tissue in patients with HFpEF. It has been reported that visceral fat is larger in patients with HFpEF than in those without HF and that its amount is associated with worse hemodynamics and reduced exercise capacity [6,7,8,22,27]. Despite the well-known obesity paradox in HF, increased visceral adiposity is associated with worse clinical outcomes in patients with HFpEF [28,29]. In accordance with these data, we found that the CT-derived visceral adipose tissue area was larger in the LVDD group than in the controls, and visceral adipose tissue area was related to worse diastolic dysfunction. These data suggest that, at least, quantitative abnormalities of visceral adiposity contribute to the underlying pathophysiology of HFpEF.

### 4.3. Limitation

The current study has important limitations. We recruited patients who were scheduled for abdominal surgery, which led to selection and referral bias. There were few patients with established HFpEF, and most had asymptomatic LVDD, which may have diluted the potential impact of the inflammatory effects of visceral adiposity and resulted in no difference in mRNA expression between the LVDD and control groups. Despite these limitations, this study was the first to provide data directly assessing qualitative abnormality of visceral adiposity in patients with significant LVDD.

## 5. Conclusions

The present preliminary data suggest that quantitative abnormalities of visceral adiposity contribute to LVDD. We found no differences in mRNA expressions of inflammatory cytokines and adipokines between patients with LVDD and controls. Further study is warranted to determine the role of qualitative abnormalities of visceral fat in patients with HFpEF.

## Figures and Tables

**Table 1:** Clinical demographics.

	Controls (n = 33)	LVDD (n = 44)	*p* Value
Age (years)	65 ± 13	73 ± 10	0.002
Female, n (%)	15 (45%)	27 (61%)	0.17
Body weight (kg)	60 ± 10	61 ± 13	0.71
Body mass index (kg/m^2^)	23.0 ± 3.4	24.0 ± 3.8	0.21
Location of primary tumor, (%) Hepatobiliary system/Pancreas/GI tract/others	24%/21%/42%/12%	14%/14%/57%/16%	0.43
***Vital signs***			
Systolic BP (mmHg)	127 ± 21	133 ± 20	0.27
Diastolic BP (mmHg)	75 ± 15	71 ± 13	0.20
Heart rate (bpm)	75 ± 13	23 ± 14	0.26
***Comorbidities***			
Hypertension, n (%)	18 (69%)	34 (85%)	0.13
Coronary artery disease, n (%)	1 (4%)	9 (23%)	0.04
Atrial fibrillation, n (%)	0 (0%)	8 (20%)	0.02
Diabetes mellitus, n (%)	7 (27%)	19 (46%)	0.11
***Medications***			
ACEIs/ARBs, n (%)	8 (31%)	18 (45%)	0.25
Beta-blocker, n (%)	1 (4%)	9 (23%)	0.04
Diuretic, n (%)	2 (8%)	7 (18%)	0.26
MRA, n (%)	0 (0%)	4 (10%)	0.11
***Laboratories***			
Hemoglobin (g/dL)	12.9 ± 1.5	12.3 ± 1.9	0.16
Creatinine (mg/dL)	0.7(0.6, 0.9)	0.8 (0.7, 1.1)	0.04
Albumin (g/dL)	4.2 ± 0.4	3.8 ± 0.5	0.0009
BNP (pg/mL)	16 (10, 28)	36 (23, 95)	0.003
White blood cell counts (×10^3^/µL)	5.6 (4.8, 6.7)	5.9 (5.0, 7.4)	0.29
Hb A1c (%)	6.1 ± 0.9	6.2 ± 0.8	0.71
GNRI (points)	105 ± 9	101 ± 11	0.07

Values are mean ± SD, median (interquartile range), or n (%). ACEIs/ARBs, angiotensin-converting enzyme inhibitors/angiotensin receptor blockers; BNP, B-type natriuretic peptide; BP, blood pressure; GI, gastrointestinal; GNRI, Geriatric Nutritional Risk Index; LVDD, left ventricular diastolic dysfunction; and MRA, mineralocorticoid receptor antagonists.

**Table 2:** Echocardiographic Findings.

	Controls (n = 33)	LVDD (n = 44)	*p* Value
***LV structure and volumes***			
LV mass index (g/m^2^)	73 ± 14	95 ± 27	<0.0001
Relative wall thickness	0.40 ± 0.06	0.43 ± 0.08	0.04
LV end-diastolic dimension (mm)	43 ± 4	46 ± 6	0.03
***LV systolic function***			
LV ejection fraction (%)	68 ± 7	65 ± 8	0.15
LV end-systolic dimension (mm)	27 ± 4	29 ± 6	0.05
Mitral s′ average velocity (cm/sec)	8.8 ± 1.3	7.4 ± 1.7	0.001
GLS (%)	21 ± 2	18 ± 4	0.0002
***LV diastolic function***			
Mitral E-wave (cm/sec)	61 ± 13	71 ± 22	0.02
Mitral average e′ velocity (cm/sec)	8.0 ± 2.5	6.6 ± 1.8	0.007
E/e′ ratio (average)	8.0 ± 1.9	11.2 ± 3.9	<0.0001
***LA structure and function***			
LA volume index (mL/m^2^)	22 (17, 27)	38 (28, 45)	<0.0001
LA reservoir strain (%)	39 ± 7	26 ± 12	0.0001
LA booster pump strain (%)	24 ± 7	18 ± 8	0.007
***Right heart***			
TRPG (mmHg)	20 ± 5	22 ± 7	0.26
eRVSP (mmHg)	23 ± 6	25 ± 7	0.18
TAPSE (mm)	21 ± 3	21 ± 5	0.91
eRA pressure (mmHg)	3 ± 0	4 ± 2	0.17

Values are mean ± SD, or median (interquartile range). eRVSP, estimated right ventricular systolic pressure; GLS, global longitudinal strain; LA, left atrial; LV, left ventricular; RA, right atrial; TAPSE, tricuspid annular plane systolic excursion; and other abbreviations as in Table 1.

**Table 3:** Computed tomography findings.

	Controls (n = 33)	LVDD (n = 44)	*p* Value
Visceral fat area (cm^2^)	112 ± 68	154 ± 106	0.04
Subcutaneous fat area (cm^2^)	117 ± 56	105 ± 59	0.34
BSA-indexed visceral fat area (cm^2^/m^2^)	66 ± 40	89 ± 53	0.04
BSA-indexed subcutaneous fat area (cm^2^/m^2^)	69 ± 34	62 ± 32	0.38

Values are mean ± SD. BSA, body surface area, and other abbreviations as in Table 1.

## Data Availability

The de-identified participant data will not be shared.

## Supplementary Materials

The following supporting information can be downloaded at: https://www.mdpi.com/article/10.3390/jcdd10060247/s1, Table S1: Primer sequences.

## References

[1] Borlaug B.A. (2020). Evaluation and management of heart failure with preserved ejection fraction. Nat. Rev. Cardiol..

[2] Obokata M., Reddy Y.N.V., Borlaug B.A. (2020). Diastolic Dysfunction and Heart Failure With Preserved Ejection Fraction: Understanding Mechanisms by Using Noninvasive Methods. JACC Cardiovasc. Imaging.

[3] Paulus W.J., Tschöpe C. (2013). A novel paradigm for heart failure with preserved ejection fraction: Comorbidities drive myocardial dysfunction and remodeling through coronary microvascular endothelial inflammation. J. Am. Coll. Cardiol..

[4] Redfield M.M. (2016). Heart Failure with Preserved Ejection Fraction. N. Engl. J. Med..

[5] Reddy Y.N.V., Rikhi A., Obokata M., Shah S.J., Lewis G.D., AbouEzzedine O.F., Dunlay S., McNulty S., Chakraborty H., Stevenson L.W. (2020). Quality of life in heart failure with preserved ejection fraction: Importance of obesity, functional capacity, and physical inactivity. Eur. J. Heart Fail..

[6] Reddy Y.N.V., Lewis G.D., Shah S.J., Obokata M., Abou-Ezzedine O.F., Fudim M., Sun J.-L., Chakraborty H., McNulty S., LeWinter M.M. (2019). Characterization of the Obese Phenotype of Heart Failure With Preserved Ejection Fraction: A RELAX Trial Ancillary Study. Mayo Clin. Proc..

[7] Sorimachi H., Obokata M., Takahashi N., Reddy Y.N.V., Jain C.C., Verbrugge F.H., Koepp K.E., Khosla S., Jensen M.D., Borlaug B.A. (2021). Pathophysiologic importance of visceral adipose tissue in women with heart failure and preserved ejection fraction. Eur. Heart J..

[8] Haykowsky M.J., Nicklas B.J., Brubaker P.H., Hundley W.G., Brinkley T.E., Upadhya B., Becton J.T., Nelson M.D., Chen H., Kitzman D.W. (2018). Regional Adipose Distribution and its Relationship to Exercise Intolerance in Older Obese Patients Who Have Heart Failure With Preserved Ejection Fraction. JACC Heart Fail..

[9] Matsubara J., Sugiyama S., Nozaki T., Sugamura K., Konishi M., Ohba K., Matsuzawa Y., Akiyama E., Yamamoto E., Sakamoto K. (2011). Pentraxin 3 is a new inflammatory marker correlated with left ventricular diastolic dysfunction and heart failure with normal ejection fraction. J. Am. Coll. Cardiol..

[10] Westermann D., Lindner D., Kasner M., Zietsch C., Savvatis K., Escher F., von Schlippenbach J., Skurk C., Steendijk P., Riad A. (2011). Cardiac inflammation contributes to changes in the extracellular matrix in patients with heart failure and normal ejection fraction. Circ. Heart. Fail..

[11] Tromp J., Westenbrink B.D., Ouwerkerk W., van Veldhuisen D.J., Samani N.J., Ponikowski P., Metra M., Anker S.D., Cleland J.G., Dickstein K. (2018). Identifying Pathophysiological Mechanisms in Heart Failure with Reduced versus Preserved Ejection Fraction. J. Am. Coll. Cardiol..

[12] Borlaug B.A., Jensen M.D., Kitzman D.W., Lam C.S.P., Obokata M., Rider O.J. (2023). Obesity and heart failure with preserved ejection fraction: New insights and pathophysiologic targets. Cardiovasc. Res..

[13] Yang H., Negishi K., Wang Y., Nolan M., Saito M., Marwick T.H. (2016). Echocardiographic screening for non-ischaemic stage B heart failure in the community. Eur. J. Heart Fail..

[14] McDonagh T.A., Metra M., Adamo M., Gardner R.S., Baumbach A., Böhm M., Burri H., Butler J., Čelutkienė J., Chioncel O. (2021). 2021 ESC Guidelines for the diagnosis and treatment of acute and chronic heart failure. Eur. Heart J..

[15] Minamisawa M., Seidelmann S.B., Claggett B., Hegde S.M., Shah A.M., Desai A.S., Lewis E.F., Shah S.J., Sweitzer N.K., Fang J.C. (2019). Impact of Malnutrition Using Geriatric Nutritional Risk Index in Heart Failure With Preserved Ejection Fraction. JACC Heart Fail..

[16] Miyake M., Morizawa Y., Hori S., Marugami N., Shimada K., Gotoh D., Tatsumi Y., Nakai Y., Inoue T., Anai S. (2017). Clinical impact of postoperative loss in psoas major muscle and nutrition index after radical cystectomy for patients with urothelial carcinoma of the bladder. BMC Cancer.

[17] Lang R.M., Badano L.P., Mor-Avi V., Afilalo J., Armstrong A., Ernande L., Flachskampf F.A., Foster E., Goldstein S.A., Kuznetsova T. (2015). Recommendations for cardiac chamber quantification by echocardiography in adults: An update from the American society of echocardiography and the European association of cardiovascular imaging. Eur. Heart J. Cardiovasc. Imaging.

[18] Kagami K., Harada T., Yoshida K., Amanai S., Kato T., Wada N., Adachi T., Obokata M. (2022). Impaired Right Atrial Reserve Function in Heart Failure with Preserved Ejection Fraction. J. Am. Soc. Echocardiogr..

[19] Shah S.J., Kitzman D.W., Borlaug B.A., Van Heerebeek L., Zile M.R., Kass D.A., Paulus W.J. (2016). Phenotype-specific treatment of heart failure with preserved ejection fraction. Circulation.

[20] Harada T., Obokata M. (2020). Obesity-Related Heart Failure with Preserved Ejection Fraction. Heart Fail. Clin..

[21] Sorimachi H., Omote K., Omar M., Popovic D., Verbrugge F.H., Reddy Y.N., Lin G., Obokata M., Miles J.M., Jensen M.D. (2022). Sex and central obesity in heart failure with preserved ejection fraction. Eur. J. Heart Fail..

[22] Ouchi N., Parker J.L., Lugus J.J., Walsh K. (2011). Adipokines in inflammation and metabolic disease. Nat. Rev. Immunol..

[23] Ikoma T., Obokata M., Okada K., Harada T., Sorimachi H., Yoshida K., Kato T., Kurosawa K., Kurabayashi M., Murakami M. (2021). Impact of Right Atrial Remodeling in Heart Failure with Preserved Ejection Fraction. J. Card. Fail..

[24] Kagami K., Takemura M., Yoshida K., Harada T., Ms H.A., Sorimachi H., Yamaguchi K., Yoshida K., Kato T., Adachi T. (2021). Pulmonary Vascular Alterations on CT Imaging and Outcomes in Heart Failure with Preserved Ejection Fraction: A Preliminary Data. J. Card. Fail..

[25] Tokitsu T., Yamamoto E., Hirata Y., Kusaka H., Fujisue K., Sueta D., Sugamura K., Sakamoto K., Tsujita K., Kaikita K. (2016). Clinical significance of pulse pressure in patients with heart failure with preserved left ventricular ejection fraction. Eur. J. Heart Fail..

[26] Tsuji K., Sakata Y., Nochioka K., Miura M., Yamauchi T., Onose T., Abe R., Oikawa T., Kasahara S., Sato M. (2017). Characterization of heart failure patients with mid-range left ventricular ejection fraction-a report from the CHART-2 Study. Eur. J. Heart Fail..

[27] Sorimachi H., Burkhoff D., Verbrugge F.H., Omote K., Obokata M., Reddy Y.N.V., Takahashi N., Sunagawa K., Borlaug B.A. (2021). Obesity, venous capacitance, and venous compliance in heart failure with preserved ejection fraction. Eur. J. Heart Fail..

[28] Tsujimoto T., Kajio H. (2017). Abdominal Obesity Is Associated With an Increased Risk of All-Cause Mortality in Patients With HFpEF. J. Am. Coll. Cardiol..

[29] Chandramouli C., Tay W.T., Bamadhaj N.S., Tromp J., Teng T.-H.K., Yap J.J.L., MacDonald M.R., Hung C.-L., Streng K., Naik A. (2019). Association of obesity with heart failure outcomes in 11 Asian regions: A cohort study. PLoS Med..

