# Supplementary material for: Pathophysiologic Contributions of Visceral Adiposity to Left Ventricular Diastolic Dysfunction
Source: J Cardiovasc Dev Dis. 2023 Jun 5;10(6):247. doi: 10.3390/jcdd10060247 (PMC10299441; doi:10.3390/jcdd10060247)
Supplement: Supplementary file 1 [file jcdd-10-00247-s001.zip › jcdd-2403746-supplementary.pdf]

Table S1: Primer sequences

	Forward (5’-3’)	Reverse (5’-3’)
βactin	CTGGCACCCAGCACAATG	CCGATCCACACGGAGTATTG
IL-6	AAGCCAGAGCTGTGCAGATGAGTA	TGTCCTGCAGCCACTGGTTC
TNFα	CTGCCTGCTGCACTTTGGAG	ACATGGGCTACAGGCTTGTCACT
CRP	AATGTGAACATGTGGGACTTTGTG	CGCCAGTTCAGGACATTAGGAC
IL-1β	CCAGGGACAGGATATGGAGCA	TTCAACACGCAGGACAGGTACAG
TGFβ	TCCTGGCGATACCTCAGCAA	GCTAAGGCGAAAGCCCTCAA
IFNγ	CTTTAAAGATGACCAGAGCATCCAA	GGCGACAGTTCAGCCATCAC
IL-2	CTTCTGTGCCTGCTGCTCATA	CTTTGGGACACTTGCTGCTG
IL-8	CACTGTGTGTAAACATGACTTCCAA	TGTGGTCCACTCTCAATCACTCTC
ADIPOQ	CTGGCTATGCTCACAGTCTCACATC	CTCTGTGCCTCTGGTTCCACAA
LEP	CCTGACTGGTGCTATAGGCTGGA	GGCTGTTCAGCTGCTGTGGTAA

ADIPOQ, adiponectin; CRP, C-reactive protein; IFN, interferon; IL, interleukin; LEP, leptin; TGF, transforming growth factor; and TNF, tumor necrosis factor.
